# Dataset of SCADA traffic captures from a medical waste incinerator with injected cyberattacks

**DOI:** 10.1016/j.dib.2025.112294

**Published:** 2025-11-20

**Authors:** Basheer Al-Duwairi, Ahmed Shatnawi, Ahmad Al-Hammouri, Mohammad Ababneh

**Affiliations:** a Department of Network Engineering & Security, Jordan University of Science and Technology, P.O. Box 3030, Irbid 22110, Jordan; b The Beacom College of Computer and Cyber Sciences, Dakota State University, 820N Washington Ave., Madison, SD 57042, USA

**Keywords:** Cybersecurity, Network traffic, Packet capture, Industrial control systems, Cyberattacks, PROFINET, OPC

## Abstract

This paper presents network traffic captures from a Supervisory Control and Data Acquisition (SCADA) system installed in a medical waste incinerator. The dataset comprises 14 daily packet capture files (day01.pcap to day14.pcap), collected over a two-week period from a Siemens S7–1500/ET200MP-based SCADA system. The total number of packets in all files is over 19 million packets. The traffic was captured using Wireshark on the Human-Machine Interface (HMI) terminal directly connected to the Programmable Logic Controller (PLC), recording network communication via the OPC and PROFINET protocols. In addition, eight traffic captures were generated by injecting relevant synthetic cyber-attack traffic into selected daily captures to simulate real-life attack scenarios, including Man-in-the-Middle (MITM), Replay, Packet Fuzzing, Command Flooding, Data Spoofing, Protocol Exploitation, Stealthy Command Injection, and SYN Flooding. Each attack-injected files contains approximately 20,000 attack packets. This dataset is invaluable to support cybersecurity research in industrial control systems (ICS), and it provides a comprehensive resource for analyzing normal SCADA behavior and for evaluating intrusion detection systems (IDS) under various common attack conditions.

Specifications Table

**Table:** 

Subject	Computer Sciences
Specific subject area	Cybersecurity in industrial control systems
Type of data	Network Traffic Data: Packet capture files contain raw network traffic from a SCADA system, including both normal operational data and synthetic attack traffic
Data collection	Data is collected over 14 days using Wireshark 4.0.3 on a Windows 10 HMI terminal directly connected to a Siemens S7–1500 PLC in a medical waste incinerator SCADA system. The captured traffic includes PROFINET/OPC over TCP (port 102) and LLDP, saved as PCAP files (day01.pcap to day14.pcap). Eight attack-injected files were generated using Scapy 2.5.0, where ∼20,000 packets per attack type (MITM, Replay, etc.) were inserted into selected capture files. No normalization was applied; raw packet data was retained. Criteria: All HMI-PLC traffic is included; non-SCADA traffic is excluded. Source: live SCADA system operation.
Data source location	Jordan University of Science and Technology, Irbid 22110, Jordan
Data accessibility	Repository name: Dataset of SCADA Traffic Captures from a Medical Waste Incinerator with Injected Cyberattacks Data Identification Number: 10.17632/vpcr4wpgfd.4 Direct URL to data: https://data.mendeley.com/datasets/vpcr4wpgfd/4 Instructions for accessing these data: None
Related research article	None

## 1 Value of the Data

• Comprehensive SCADA Traffic Representation: This dataset provides a 14-day continuous capture of live network traffic from a Siemens S7–1500-based SCADA system operating a medical waste incinerator at the Jordan University of Science and Technology. With over 19 million packets, it offers a real-world representation of PROFINET and OPC communications in an operational industrial control system (ICS). Researchers can use this data to study ICS behavior under normal operations, understand communication patterns, and model baseline system performance.
• Diverse Attack Scenarios: The dataset includes eight attack-injected captures, each containing approximately 20,000 synthetic packets representing commonly observed cyber threats such as Man-in-the-Middle (MITM), Replay, Packet Fuzzing, Command Flooding, SYN Flooding, Stealthy Command Injection, Protocol Exploitation, and Data Spoofing. These scenarios provide a foundation for:
  ○ Developing and benchmarking intrusion detection systems (IDS) and anomaly detection frameworks,
  ○ Evaluating cyber-resilience of SCADA protocols under realistic adversarial conditions, and
  ○ Simulating controlled attack conditions for training security operations teams.
• Machine Learning Model Development: Researchers and practitioners can leverage this data for feature extraction (e.g., packet sizes, S7comm codes, timing intervals) to build, train, and evaluate machine learning (ML) and deep learning (DL) models for ICS-specific anomaly detection. Both supervised and unsupervised approaches are supported, making the dataset ideal for:
  ○ Comparing IDS detection rates under varied attack scenarios,
  ○ Building predictive models that detect subtle, stealthy manipulations in SCADA traffic, and
  ○ Establishing standardized benchmarks for cybersecurity tool evaluation.
• Interdisciplinary Applications: While designed for cybersecurity research, this dataset also serves multiple disciplines:
  ○ Healthcare Waste Management: Understanding vulnerabilities in medical waste incineration systems, which are critical for environmental safety and public health,
  ○ Environmental Protection: Analyzing operational patterns to evaluate process safety and mitigate risks in environmentally sensitive facilities, and
  ○ Industrial Automation and Control: Providing real-world data for engineers to assess protocol efficiency and system reliability.
• Policy and Regulatory Implications: As critical infrastructure systems increasingly face cyber threats, this dataset provides a data-driven foundation for developing standards, guidelines, and compliance frameworks. Policymakers, regulatory bodies, and certification authorities can leverage this data to:
  ○ Define minimum security baselines for SCADA systems in healthcare and environmental sectors,
  ○ Establish testing benchmarks for ICS/SCADA security solutions, and
  ○ Support incident response planning and resilience assessment at national and organizational levels.
• Comparison with Other ICS/SCADA Datasets: Compared to widely used datasets such as SwaT [1], and WADI [2], this dataset offers unique advantages:
  ○ Unlike SWaT and WADI, which are testbed-based environments, our dataset originates from a fully operational medical waste incinerator, providing authentic communication patterns,
  ○ Unlike many power system datasets, this dataset focuses on healthcare-related critical infrastructure, making it especially relevant for cross-sector risk assessment, and
  ○ It uniquely captures PROFINET and OPC traffic from Siemens S7–1500/ET200MP devices, which are underrepresented in existing open datasets.
• Support for Reproducibility through Metadata: To enhance reproducibility and ease of reuse, we provide detailed metadata for every PCAP file, including:
  ○ Packet counts,
  ○ Average packets per second (PPS),
  ○ File sizes,
  ○ Attack injection intervals and strategies,
  ○ MD5 checksums for integrity verification, and
  ○ Python scripts used for both traffic sanitization and attack generation
    This additional information enables researchers to replicate experiments, benchmark tools, and extend the dataset for broader applications.

• Real-World Industrial Context: Unlike many existing ICS datasets collected from simulated environments, this dataset originates from a fully operational medical waste incinerator. It offers authentic communication dynamics, enabling researchers to:
  • Identify protocol-specific vulnerabilities in PROFINET and OPC implementations,
  • Understand the impact of cyberattacks on mission-critical healthcare operations, and
  • Develop security hardening strategies for similar industrial setups.

## 2 Background

Supervisory Control and Data Acquisition (SCADA) systems play a crucial role in managing critical infrastructure such as power systems, water management systems, medical waste incinerators, etc., However, as these systems connect to open networks, they become more exposed to cyber threats and more attractive targets of cyberattacks. Therefore, providing realistic datasets from an operational SCADA environment is fundamental in developing efficient AI-based intrusion detection mechanisms. Past studies have highlighted SCADA’s weaknesses to the attacks of Man-in-the-Middle [3], replay [4], and protocol exploitation [5]. Our dataset comes straight from a real-world Siemens S7–1500/ET200MP-based SCADA system running a medical waste incinerator that supports both healthcare and environmental needs. The dataset includes PROFINET and OPC communications, thus giving a clear picture of normal operations and specific protocol behaviors [6]. Since such a facility is so critical, any disruption could lead to serious public health and environmental consequences.

Drawing on ICS security frameworks [7,8], this dataset includes both normal traffic and simulated attacks to help develop anomaly-based intrusion detection systems (IDSs). It addresses the scarcity of public datasets capturing Siemens S7–1500 traffic under varied conditions [9] and complements existing research such as the SWaT testbed [10]. Over 14 days, >19 million packets were recorded using Wireshark 4.0.3. Synthetic attacks—including MITM, replay, fuzzing, command flooding, spoofing, exploitation, stealthy injection, and SYN flooding—were designed to emulate real-world adversarial scenarios [11].

## 3 Data Description

The dataset is organized into a single repository [12] containing four main directories: Normal_Traffic, Attack_Injected_Traffic, scripts, and metadata, as described below.

• Normal_Traffic/
  Contains 14 packet capture files (day01.pcap to day14.pcap) representing normal SCADA network traffic collected continuously over 14 consecutive days from the medical waste incinerator’s Siemens S7–1500-based control system.
• Attack_Injected_Traffic/
  Contains eight packet capture files where synthetic cyberattacks were injected into selected baseline captures. Each file name specifies the attack type and the day number of the corresponding normal traffic file used for injection. Each capture file has an approximate size of 160 MB, depending on the attack type and duration.
  Examples:
  ○ traffic_with_syn_flooding_attack04.pcap → SYN flooding injected into day04.pcap.
  ○ traffic_with_stop_plc_attack10.pcap → STOP PLC commands injected into day10.pcap.
• scripts/
  Includes all Python scripts used to generate the attack-injected PCAP files and to sanitize sensitive information. These scripts allow researchers to replicate or extend the attack generation and data preprocessing workflows.
  Among these, scada_pcap_sanitizer.py is specifically designed to anonymize sensitive identifiers such as IP addresses, MAC addresses, and VLAN IDs to protect the integrity and confidentiality of the original SCADA network.
• metadata/
  Contains auxiliary files that enhance reproducibility and data integrity verification:
  ○ per_file_statistics.csv → Provides detailed metadata for each capture file, including packet counts, average packets per second (PPS), file sizes, and injection intervals.
  ○ checksums.md5 and checksums.sha256 → Offer integrity hashes for all PCAP files, enabling verification after download.

This structured organization ensures the dataset is intuitive to navigate, while maintaining traceability between original captures, attack-injected variants, and supporting scripts. Researchers can seamlessly reproduce the attack injection process, verify dataset integrity, and integrate the data into experimental workflows.

**Folder Structure Overview:** 

**File Naming Convention**

To ensure consistency, the naming scheme of the attack-injected PCAP files follows the structured format:

traffic_with_<attack_type>_attackXX.pcap

• <attack_type> → Indicates the specific type of cyberattack injected into the normal SCADA traffic (e.g., syn_flooding, stop_plc, replay).
• XX → Represents the day number of the base capture file used for generating the attack-injected variant.

For example:

• traffic_with_syn_flooding_attack04.pcap → A SYN flooding attack injected into the day04.pcap normal traffic file.
• traffic_with_stop_plc_attack10.pcap → A STOP PLC command attack injected into the day10.pcap normal traffic file.

This convention ensures that researchers can easily trace each attack-injected capture back to the corresponding normal traffic baseline used during generation, thus improving both reproducibility and usability.

## 4 Experimental Design, Materials and Methods

### 4.1 Data acquisition

The dataset was acquired via a two-phase process: (i) the capture of live SCADA traffic, and (ii) the generation of attack-injected traffic of a given live capture. The live traffic collection was conducted over 14 consecutive days at a medical waste incinerator SCADA system located at the Jordan University of Science and Technology. The SCADA system operated continuously within the medical waste incinerator, processing waste materials 24 h a day under standard operational protocols. The hardware setup of the incinerator comprises a Siemens S7–1500/ET200MP PLC with a 1.5 GHz processor and 1 GB of RAM, and an HMI with an Intel i5–8500 processor, 8 GB of RAM, and a Gigabit Ethernet interface.

The network was an isolated Ethernet segment connecting the PLC, HMI, and an IO gas cabinet, with no external internet connection to prevent interference. Based on the operational load, the daily traffic captures we recorded ranged from about 1.3 to 1.5 million packets each day. The attack injection process was performed offline on a separate macOS workstation after the live capture phase, processing one base file at a time to generate each attack-injected file. The isolated network ensured that captured traffic reflected only SCADA-specific communications, with physical security maintained at the facility to prevent unauthorized access during the experiment. Given the sensitivity of the medical waste incinerator’s SCADA network, a custom Python-based PCAP sanitization tool was developed, leveraging Scapy for packet parsing and manipulation, TQDM for real-time progress tracking, and multiprocessing to handle the extremely large traffic files efficiently.

The tool was carefully designed to perform consistent pseudonymization of sensitive identifiers. Specifically, it replaced all MAC addresses, IPv4/IPv6 addresses, and VLAN IDs with pseudonymous but uniform values across the entire dataset. This ensured that while the actual identifiers were concealed, the logical relationships between devices, communication paths, and roles within the network remained intact and analytically meaningful. For instance, if a specific PLC communicated frequently with a Human-Machine Interface (HMI), these interactions would still be visible in the sanitized dataset, but without exposing the real addresses or device identifiers. In addition to anonymizing network-level identifiers, the tool removed sensitive metadata embedded in packet headers and payloads that could otherwise reveal details such as PLC hardware models, HMI configurations, and the network topology of the SCADA environment. These measures were implemented to prevent adversaries from inferring vulnerabilities or operational characteristics of the system while still preserving the dataset’s value for traffic analysis, intrusion detection research, and machine learning applications.

A critical design principle of the tool was integrity preservation. Care was taken to maintain the original packet timing, protocol hierarchies, and communication sequence flows to ensure that the dataset accurately reflected the real operational dynamics of the SCADA system. This means that statistical properties, such as traffic volumes, latency patterns, and command-response behaviors, remained unchanged after sanitization, allowing researchers to analyze authentic SCADA traffic patterns without compromising the system’s confidentiality.

### 4.2 Software and tools

• **Wireshark 4.0.3:** Installed on the HMI to capture live traffic and is configured to capture every Ethernet frame on the connection between the HMI and the PLC.
• **Python 3.12:** This is used for writing scripts to perform the anonymization and to simulate attacks. The scripts were run on a separate macOS machine after the initial capture.
• **Scapy 2.5.0:** This is used to create and insert synthetic attack packets into chosen PCAP files.
• **Operating Systems:** We used Windows 10 on the HMI for capturing live traffic, and macOS Sonoma 14 on a separate workstation to generate attacks and run scripts.

Additional utilities include Npcap 1.70 used for Wireshark packet capture on Windows and standard macOS networking tools for file transfer and validation.

### 4.3 Synthetic attack generation

**Fig. 1:**
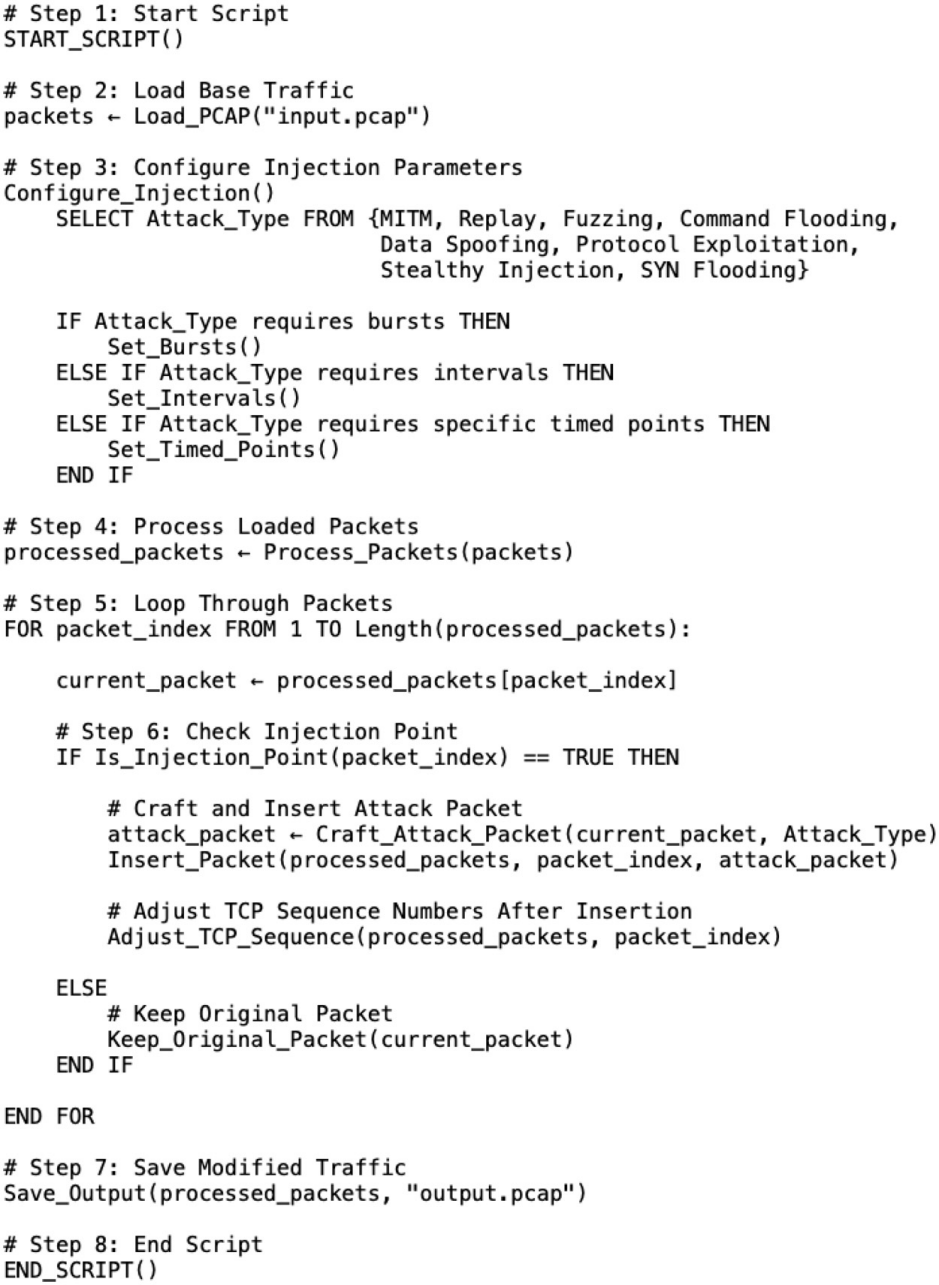
Pseudocode illustrating the automated synthetic attack injection process.

The pseudocode depicted in Fig. 1 represents the synthetic attack generation process. The procedure begins by loading a benign SCADA PCAP file and configuring the injection parameters, including the attack type and timing strategy (bursts, intervals, or timed points). The loaded packets are then processed and iterated over sequentially. At each step, the algorithm checks whether the current packet is an injection point. If it is, an attack packet is crafted based on the selected attack type and parameters, and is inserted into the traffic stream at set intervals or in bursts at specific spots. This step is similar to methods used in packet fuzzing and stealthy injection techniques [9] to keep the attacks realistic. After insertion, the TCP sequence numbers of subsequent packets are adjusted to maintain communication integrity. Otherwise, the original packet is kept unchanged. Finally, the modified packet file is saved as a new PCAP file, ready for further analysis and testing.

#### 4.3.1 Script files

Eight Python scripts were developed using Scapy 2.5.0 to generate attack-injected traffic, each corresponding to a specific attack type as follows:

• script_mitm.py: Injects 20,000 MITM attack packets.
• script_replay.py: Injects 20,000 replay attack packets.
• script_fuzzing.py: Injects 20,000 fuzzing attack packets in 5 bursts of 4000.
• script_command_flooding.py: Injects 20,000 command flooding packets in 3 bursts of ∼6667.
• script_data_spoofing.py: Injects 20,000 data spoofing packets.
• script_s7comm_exploit.py: Injects 20,000 protocol exploitation packets.
• script_stealthy_command_injection.py: Injects 20,000 stealthy injection packets every 55 packets.
• script_syn_flooding.py: Injects 20,000 SYN flooding packets in 5 bursts of 4000.

### 4.4 Injected attack description

We crafted eight different types of cyberattacks and added them to the daily SCADA traffic captures using Scapy 2.5.0, with each attack type contributing around 20,000 packets. Our goal was to create realistic threat scenarios in a SCADA environment, specifically targeting the Siemens S7–1500 PLC and its communication with the HMI using PROFINET and OPC protocols over TCP port 102. Importantly, we made sure the original traffic stayed untouched while weaving in these synthetic attack packets, mimicking disruptions that could realistically happen in a medical waste incinerator system.

• *Man-in-the-Middle (MITM):* The study by Yang et al. [11] highlighted several vulnerabilities in smart grid SCADA systems, demonstrating how attackers could successfully carry out man-in-the-middle (MITM) attacks. They used a testbed to intercept PROFINET traffic, showing these attacks are not just theoretical. In our study, we simulated an attacker secretly intercepting and tampering with HMI-PLC communications. Using a script called script_mitm.py, we adjusted TCP sequence and acknowledgment numbers to sneak into the session without raising alarms. Also, source and destination IP addresses were spoofed to blend in as a legitimate device. In addition, S7comm payloads—such as setpoint values or control commands—were altered, and these tampered packets were injected at a rate of approximately one for every 100 normal packets. This mimics a real attacker passing along fake instructions, which could lead to unauthorized changes in the system’s operations.
• *Replay:* Replay attacks in ICS were thoroughly examined in [4], which revealed how manipulating timestamps can bypass basic sequence checks in S7–1500 systems. In our case, we copied legitimate packets from a base file, day05.pcap, and reinserted them using script_replay.py. The script picks out PROFINET/OPC commands—such as read or write requests—adjusts their timestamps to fit the current session, and adds 20,000 of these duplicated packets over the capture period. This simulates an attacker resending valid commands to interfere with the PLC, such as repeatedly triggering a valve to open or close.
• *Packet Fuzzing:* We tested the PLC’s resilience by injecting random payloads with script_fuzzing.py. This script creates 20,000 packets with malformed S7comm headers, such as invalid function codes, or random data payloads between 15 and 50 bytes. We added them in five bursts of 4000 packets each, at positions (i.e., indices or lines) 200,000, 400,000, 600,000, 800,000, and 1000,000. These bursts mimic sporadic fuzzing attempts that could crash or confuse the PLC’s protocol parser. Liu et al. [13] studied fuzzing S7comm in SCADA systems and found that malformed packets can exploit weak input validation, which formed the basis of our implementation.
• *Command Flooding:* We conducted this attack by flooding the PLC with high-rate command packets using script_command_flooding.py. The script generates 20,000 S7comm write commands—such as repeated memory writes—in three bursts of about 6667 packets each, at positions 300,000, 600,000, and 900,000. This flood aims to consume the PLC’s processing power, mimicking a denial-of-service (DoS) scenario that could slow down critical responses. Research in [14] looked into this type of attacks in ICS networks and confirmed its ability to disrupt Siemens PLC operations.
• *Data Spoofing:* We forged sensor data packets using script_data_spoofing.py, injecting 20,000 packets with false values—such as reporting a temperature of 500 °C instead of 200 °C—within PROFINET frames. These were evenly spread out, with one spoofed packet for every 75 normal ones. This mimics an attacker feeding misleading data to trick the system into making bad decisions, such as triggering an unnecessary shutdown. Bhattar and Pindoriya [15] explored cyber threats to smart grids, pointing out the dangers of data spoofing, false data injections, and denial-of-service attacks, especially how they exploit vulnerabilities in state estimators within ICT-enabled setups.
• *Protocol Exploitation (S7comm Exploit):* We injected packets that exploit S7comm protocol weaknesses using script_s7comm_exploit.py. This script creates 20,000 packets with flawed headers, such as oversized parameter fields, or unauthorized commands, such as memory read requests beyond allowed limits, spread evenly across the capture. This tests how the PLC handles protocol flaws, which could let an attacker gain unauthorized access. Hui et al. [5] investigated S7comm exploitation techniques, pinpointing vulnerabilities in Siemens PLC firmware that we studied here.
• *Stealthy Command Injection*: Subtle command packets were injected with script_stealthy_command_injection.py, adding 20,000 valid S7comm commands—such as tiny setpoint changes of 0.1 °C—every 55 packets. These blend into normal traffic to avoid detection, simulating a sneaky attacker slowly altering the system, such as gradually raising the incinerator’s temperature. Alsabbagh et al. [16] studied stealthy injections in ICS and showed how small low-rate command changes can bypass traditional intrusion detection systems.
• *SYN Flooding:* We simulated a denial-of-service attack by injecting TCP SYN packets with script_syn_flooding.py. The script generates 20,000 packets with random source ports (1024–65,535) and SYN flags set, delivered in five bursts of 4000 packets at positions 250,000, 500,000, 750,000, 1000,000, and 1250,000. This flood tries to overload the PLC’s connection table, disrupting HMI-PLC communication. Arifin et al. [17] shared a SCADA dataset using the IEC 60,870–5–104 protocol, featuring attack scenarios such as SYN floods, which underscored the vulnerability of RTUs in electricity distribution networks.

It’s worth noting that each attack was added to a separate copy of a base PCAP file, e.g., day05.pcap, so the original traffic remained intact while we layered in the synthetic attack data. We adjusted TCP sequence numbers as needed to keep the session flowing smoothly, and the attack packets were timestamped to match the original capture timeline.

### 4.5 Attack injection analysis

Table 1 summarizes the key parameters of the synthetic attack injections applied to the SCADA traffic captures. It details the attack type, injection strategy, number of injected packets, average payload size, injection interval, and direction.

**Table 1:** Attack injection summary.

Attack Type	Injection Strategy	Total Packets Injected	Avg Payload Size (bytes)	Injection Interval	Direction
Stop PLC Attack	Inject Stop command every 55 packets	20,000	24	55 packets	HMI -> PLC
Command Flooding	Burst injections at positions [300k,600k,900k] with alternating read/write packets	20,000	34	Burst	HMI -> PLC
Data Spoofing	Inject spoofed PLC response every 55 packets	20,000	23	55 packets	PLC -> HMI
SYN Flooding (DDoS)	Burst injections at positions [200k,400k,600k,800k,1000k] with 4000 SYN packets per burst	20,000	0	Burst	HMI -> PLC
Fuzzing Attack	Burst injections at positions [200k,400k,600k,800k,1000k] with randomized fuzz packets	20,000	48	Burst	HMI -> PLC
MITM / Stealthy Injection	Inject a subtle S7comm write packet every 22,000 packets	50	33	22000 packets	HMI -> PLC
Protocol Exploitation	Inject exploit packets every 55 packets with 1000-byte padding	20,000	1031	55 packets	HMI -> PLC
Replay Attack	Replay a captured S7comm packet every 55 packets	20,000	37	55 packets	HMI -> PLC
Stealthy Command Injection	Inject stealthy S7comm write packet every 55 packets	20,000	37	55 packets	HMI -> PLC

The attack scenarios vary in injection frequency and payload size, demonstrating both high-frequency attacks and stealthy, low-volume interventions in attack-injected traffic. This guarantees diversity in benign and attack-injected traffic files.

## Limitations

The data collection was limited to the HMI-PLC interface, potentially missing other network segments. The dataset size varies slightly per file (1.3–1.5 million packets), reflecting operational differences. While background LLDP traffic complicates attack injection alignment, manual burst position adjustments risked misalignment. Finally, the focus on a single Siemens S7–1500 system may limit generalizability.

## Ethics Dtatement

The authors have read and followed the ethical requirements for publication in *Data in Brief* and confirm that the current work does not involve human subjects, animal experiments, or any data collected from social media platforms.

## CRediT Author Statement

**Basheer Al-Duwairi:** Funding acquisition, Conceptualization, Methodology, Supervision, Project administration, Data curation, Writing — original draft, and Writing — review and editing; **Ahmed Shatnawi:** Funding acquisition, Conceptualization, Methodology, Software, Validation, and Writing — original draft; **Ahmad Al-Hammouri**: Funding acquisition, Conceptualization, Methodology, Writing — original draft, Writing — review and editing, and Visualization; **Mohammad Ababneh**: Investigation, Resources, and Writing — review and editing.

## Data Availability

Mendeley DataSCADA Traffic Dataset from a Medical Waste Incinerator with Injected Cyber Attacks (Original data).
